# The effects of deprivation, age, and regional differences in COVID-19 mortality from 2020 to 2022: a retrospective analysis of public provincial data

**DOI:** 10.1186/s12889-024-21031-5

**Published:** 2025-01-14

**Authors:** Anqi A. Chen, Elizabeth M. Renouf, Charmaine B. Dean, X. Joan Hu

**Affiliations:** 1 Department of Statistics and Actuarial Science, Simon Fraser University, Burnaby, BC V5A 1S6 Canada; 2 Department of Civil Engineering, University of Ottawa, Ottawa, ON K1N 6N5 Canada; 3 Department of Statistics and Actuarial Science, University of Waterloo, Waterloo, ON N2L 3G1 Canada

**Keywords:** SARS-CoV-2 infection, Generalized additive models, COVID-19 vaccines, Public reporting of health care data, COVID-19 hospitalizations, Random effects

## Abstract

**Background:**

Coronavirus disease (COVID-19) quickly spread around the world after its initial identification in Wuhan, China in 2019 and became a global public health crisis. COVID-19 related hospitalizations and deaths as important disease outcomes have been investigated by many studies while less attention has been given to the relationship between these two outcomes at a public health unit level. In this study, we aim to establish the relationship of counts of deaths and hospitalizations caused by COVID-19 over time across 34 public health units in Ontario, Canada, taking demographic, geographic, socio-economic, and vaccination variables into account.

**Methods:**

We analyzed daily data of the 34 health units in Ontario between March 1, 2020 and June 30, 2022. Associations between numbers of COVID-19 related deaths and hospitalizations were explored over three subperiods according to the availability of vaccines and the dominance of the Omicron variant in Ontario. A generalized additive model (GAM) was fit in each subperiod. Heterogeneity across public health units was formulated via a random intercept in each of the models.

**Results:**

Mean daily COVID-19 deaths increased quickly as daily hospitalizations increased, particularly when daily hospitalizations were less than 20. In all the subperiods, mean daily deaths of a public health unit was significantly associated with its population size and the proportion of confirmed cases in subjects over 60 years old. The proportion of fully vaccinated (2 doses of primary series) people in the 60 + age group was a significant factor after the availability of the COVID-19 vaccines. The deprivation index, a measure of poverty, had a significantly positive effect on COVID-19 mortality after the dominance of the Omicron variant in Ontario. Quantification of these effects was provided, including effects related to public health units.

**Conclusions:**

The differences in COVID-19 mortality across health units decreased over time, after adjustment for other covariates. In the last subperiod when most public health protections were released and the Omicron variant dominated, the least advantaged group might suffer higher COVID-19 mortality. Interventions such as paid sick days and cleaner indoor air should be made available to counter lifting of health protections.

**Supplementary Information:**

The online version contains supplementary material available at 10.1186/s12889-024-21031-5.

## Background

 Coronavirus disease (COVID-19) is an infectious disease caused by the severe acute respiratory syndrome coronavirus 2 (SARS-CoV-2). The virus was initially identified in Wuhan, China in December 2019 and quickly spread around the world. Hospitalizations and deaths are important outcomes that quantify the severity of COVID-19 [1, 2]. Many studies have investigated the association of certain risk factors with COVID-19 mortality and hospitalizations or in-hospital mortality [3–7]. However, the relationship between COVID-19 deaths and hospitalizations remains insufficiently explored. Understanding this relationship can provide valuable insights into healthcare system capacity and disease progression. For example, a disproportionate number of deaths compared to hospitalizations may indicate overwhelming hospital burdens; a rise in hospitalizations may serve as an early warning on a potential increase in deaths. This study can bridge the gap in the literature. Furthermore, the association of deaths with hospitalizations likely varies over time and is dependent upon multiple factors such as public health policy and implementation [8–10], population immunity from vaccination and previous infection [11, 12], material deprivation [13], and characteristics of variants of concern [14, 15].

Ontario is the southernmost and most populous province in Canada. There are 34 public health units in Ontario, which provide the corresponding regions with health education, disease control and other related services [16]. Public Health Ontario, a government organization, offers publicly accessible COVID-19 data summarized by public health unit and date. During the pandemic, similar platforms have provided open-access, real-time aggregate data to track disease spread across regions [17, 18]. Aggregate COVID-19 data have shorter reporting time compared to individual-level data, making them more practical and efficient for timely decision-making [19]. Thus, it is essential to develop appropriate statistical methods to analyze aggregate data. In literature, some studies have been conducted based on aggregate COVID-19 data [8, 20–23].

This study aims to establish the relationship of counts of COVID-19 related deaths and hospitalizations across the 34 public health units in Ontario over different time periods, using publicly available aggregate data. During the study period between March 1, 2020 and June 30, 2022, there were a total of 13,435 deaths and 49,805 hospitalizations in Ontario; Toronto, the most populous city in Ontario, contributed the most with 31.8% of the deaths and 29.4% of the hospitalizations. Approximately 91.9% of the deaths and 67.3% of the hospitalizations occurred in people over 60 years old.

We apply generalized additive models [24] over different subperiods to provide great flexibility in modeling. A generalized additive model (GAM) can be regarded as a generalized linear model (GLM) whose systematic component is replaced by a sum of unspecified smooth functions of covariates. The smooth functions can capture nonlinear covariate effects on a response. GAMs are a commonly used tool for analyzing the effect of air pollution or other exposures on health outcomes such as hospitalizations and mortality [25, 26]. Different GAM models have been applied to predict COVID-19 related mortality risk in Toronto, Canada. For example, Feng, Kephart, and Wagner [27] compared the predictive performance of a GAM with some tree-based (e.g., random forest and extreme gradient boosting) and regression-based (e.g., LASSO) machine learning methods, finding comparable predictive performance for GAM, LASSO, logistic regression, and extreme gradient boosting. Feng [28] proposed a spatial-temporal GAM to model the COVID-19 mortality rate in Toronto, Canada, using a tensor product smoother of geographic location and time, to deal with the potential spatial correlation over time. Izadi [29] considered GAMs with smooth functions of weekly, biweekly, and monthly indices to explore the corresponding patterns of death rates in Canada and three Canadian provinces separately.

## Methods

Our study goal is to establish the relationship of deaths and hospitalizations caused by COVID-19 over time across 34 public health units in Ontario, Canada while taking demographic, geographic, socio-economic, and vaccination variables into consideration. Our statistical analysis is undertaken at the level of public health unit.

### Study population

Ontario, the most populous province and the second largest by area in Canada, had a population of approximately 13.4 million living in a total land area of 908,699 square kilometres in 2016 [30]. Geographically, Ontario shares its southern border with the United States and is the home to both the nation’s capital, Ottawa, and the largest city, Toronto. According to the 2016 Census [30], the average age of Ontario’s population is 41.0 years (median: 41.3 years), with 18.9% of residents aged 65 or older. Females constitute 51.2% of the population. In the labour force, leading occupational categories are sales and service occupations (22.9%) and business, finance, and administration occupations (15.7%).

Ontario is divided into 34 public health units, each defined by specific geographical boundaries. The health units offer health programs and disease prevention information and services to their respective populations [16]. The units vary dramatically in population density and geographic size. For example, Toronto Public Health, the most populous health unit, has nearly twice the population of the next largest unit, Peel Public Health [30].

### Data and data source

We extracted the COVID-19 data between March 1, 2020 and June 30, 2022 from the COVID-19 Data Tool [31]. The tool provided daily COVID-19 outcomes (counts and rates of confirmed cases, hospitalizations, and deaths) and vaccination data (total numbers of individuals getting fully vaccinated, receiving the first and the second booster doses, and more). These data were summarized by sex (female and male), age groups (0–4, 5–11, 12–19, 20–39, 40–59, 60–79, and 80+), and public health units.

In the dataset, deaths were recorded as COVID-19 mortality only if the individual had not fully recovered from COVID-19 before death and the death occurred within the follow-up period. Consequently, the deaths in our dataset might be under-reported. Daily new hospitalizations were grouped by episode date, representing the estimated date of disease onset. Since the number of deaths on a given day was likely associated with the hospitalizations in the preceding days, we utilized a surrogate for this lagged effect, the average of daily hospitalizations over the last 14 days (including the current day, referred to as *14 day.rolling.average.hospitalizations*). Additionally, the 2020 population size for each health unit (*population*) was obtained from Statistics Canada [30]. Using the available COVID-19 data, we derived several daily covariates, including the proportions of confirmed cases in males (*proportion.cases.male*) and in subjects over 60 years old (*proportion.cases.over60yrs*), as well as the proportions of fully vaccinated subjects (i.e., receiving two doses, labelled as *proportion.of.fully.vaccinated.over60yrs*), subjects with 1 booster dose (*proportion.of.1st.booster.over60yrs*), and subjects with 2 booster doses (*proportion.of.2nd.booster.over60yrs*) in the over 60-year-old population. We focused on people aged 60 and older since they accounted for the majority of COVID-19 hospitalizations and deaths. Moreover, we included the 2016 marginalization index from Public Health Ontario [32] as a constant measure for each health unit. Since some marginalization index scores were correlated, we only used the deprivation index (*deprivation*), an economic measure reflected by indicators of income, quality of housing, family structure, and education level [33].

In the following analysis, we considered the daily count of COVID-19 deaths as the response variable and all the variables with italicized covariate names as potential predictors. Given prior studies highlighting associations between COVID-19 outcomes and demographic, vaccination, and socioeconomic factors [11–13, 23], we applied forward selections to allow the data to identify the important covariates included in the final models.

### Statistical analysis

We divided the entire study period into three subperiods according to the availability of COVID-19 vaccines and the dominance of the Omicron variant: March 1, 2020 to December 13, 2020 (before the availability of COVID-19 vaccines), December 14, 2020 to December 14, 2021 (after vaccine availability but before at least 50% of sampled genomes identified as Omicron), and December 15, 2021 to June 30, 2022 (after the dominance of the Omicron variant). For each subperiod, we fitted a GAM with the daily number of deaths as the response variable; the potential explanatory variables were the 14-day rolling average of hospitalizations, days from March 1, 2020, population size of each health unit in Ontario, the daily proportions of reported cases in males and in subjects over 60 years old, the deprivation index, the daily proportions of people getting fully vaccinated, receiving 1 booster dose, and receiving 2 booster doses in the 60 + age group. In the models, we also included random intercepts to account for the heterogeneity across the 34 health units. The Akaike information criterion (AIC) was used to implement a forward variable selection among those explanatory variables in each GAM. The initial model in the variable selection contained the random intercept for health units, and a logarithm of the population size. In the forward selection, each selected covariate was involved in the model as a smooth function or a linear/parametric term; which one to use depended on whether the smooth function had a significant (*p*-value < 0.05) effect on the response. The number of basis functions in each spline smoothing could be chosen by adjusted R-square/AIC. The forward selection stopped if including more explanatory variables did not significantly improve the model and no more explanatory variables had significant impacts on COVID-19 mortality.

Let $\:{Y}_{st}$ denote the number of deaths due to COVID-19 in health unit *s* on day *t*, where $\:s=1,\:\cdots\:,\:34$, for three subperiods indexed by *p* where $\:t=1,\:\cdots\:,\:288$ ($\:t=\:1$ denotes March 1, 2020) for *p*=1, $\:t=289,\:\cdots\:,\:654$ for *p*=2, and $\:t=655,\:\cdots\:,\:852$ for *p*=3. Since the deaths are over-dispersed with respect to a Poisson distribution, we assume that $\:{Y}_{st}$ follows a negative binomial distribution with mean $\:E\left[{Y}_{st}|{X}_{st},{Z}_{st}\right]={{\upmu\:}}_{st}$, $\:{X}_{st}\:\text{a}\text{n}\text{d}\:{Z}_{st}$ represent the covariates in parametric/linear terms and smooth functions, respectively. The selected GAM for subperiod *p* can be expressed as

$$\:\text{l}\text{o}\text{g}\left({{\upmu\:}}_{st}\right)={{{\upbeta\:}}_{0}}^{\left(p\right)}+{{b}_{s}}^{\left(p\right)}+\sum\limits_{j=1}^{{J}_{p}}{{{\upbeta\:}}_{j}}^{\left(p\right)}{x}_{jst}+\sum\limits_{k=1}^{{K}_{p}}{f}_{k}^{\left(p\right)}\left({z}_{kst}\right),$$

where the random intercept $\:b_s^{\left(p\right)}\sim N\left(0,{\sigma\:}_p^2\right)$ for $\:p=1,\:2,\:3$, $\:{f}_{k}^{\left(p\right)}\left(\cdot\:\right)$ represents a smooth function, and $\:{{\beta\:}_{0}}^{\left(p\right)}$ and $\:{{{\upbeta\:}}_{j}}^{\left(p\right)}$ denote the fixed components of the intercept and the regression. The counts $\:{J}_{p}$ and $\:{K}_{p}$ are respectively the numbers of linear terms and smooth functions in the model for subperiod *p*. We assume that all the health units are independent with each other, and the observations over time within a health unit are independent conditional on the random intercept. We will discuss this assumption later. Details on implementation of the GAM in R (Version 4.0.2) are given in Appendix 1.

## Results

**Fig. 1:**
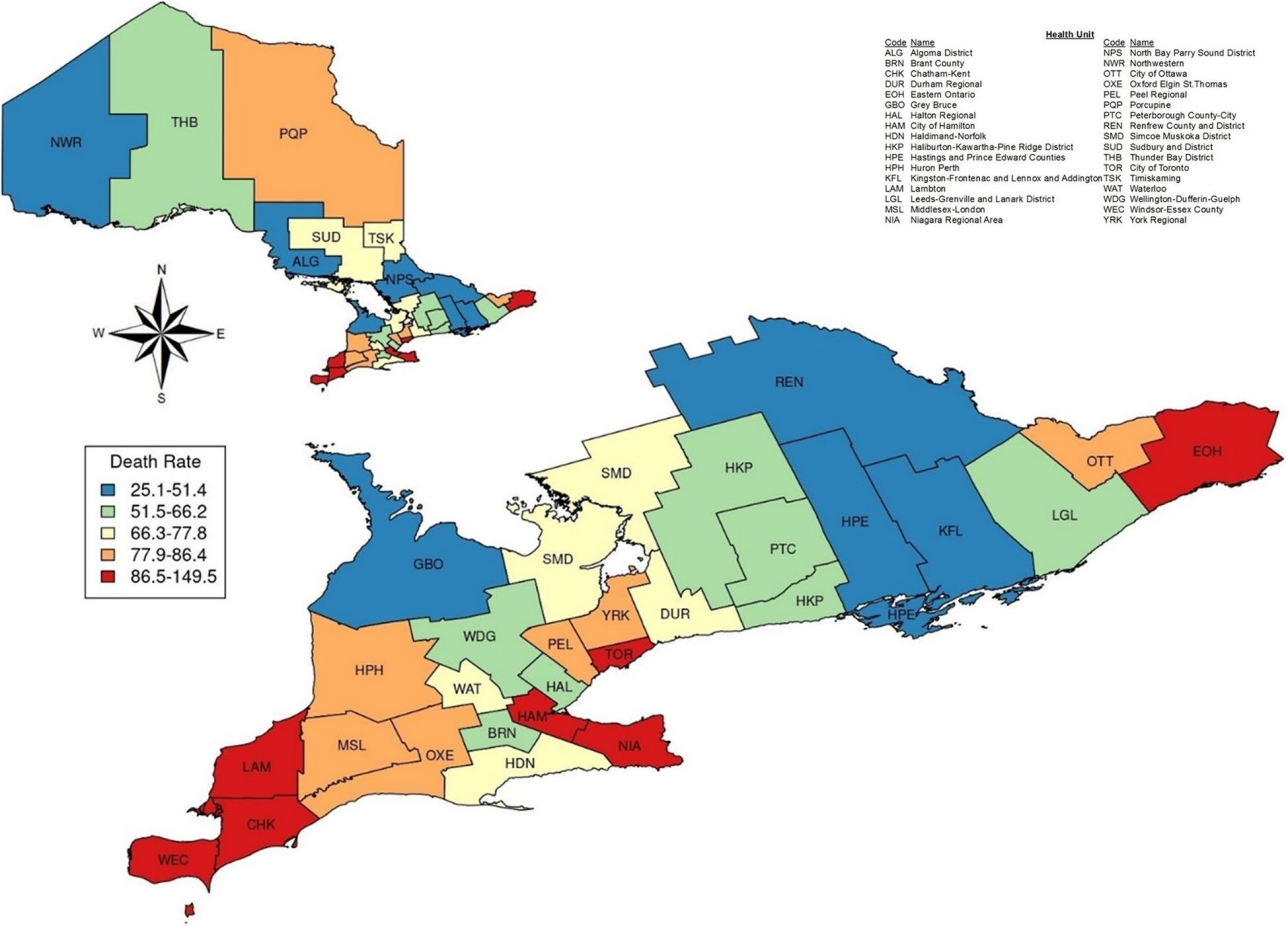
Death rates of Ontario health units during the study period (per 100,000 population)

Figure 1 [34] shows a map of the regional health units, colour-coded by the death rate per 100,000 population over the entire study period. The highest death rates are seen in Toronto (TOR, with the largest population in Ontario) and in many health units that contain border crossings with the United States and their neighbours. The busiest land border crossings in Ontario are in Windsor (WEC), Sarnia (LAM), and Fort Erie (NIA; [35]).

**Fig. 2:**
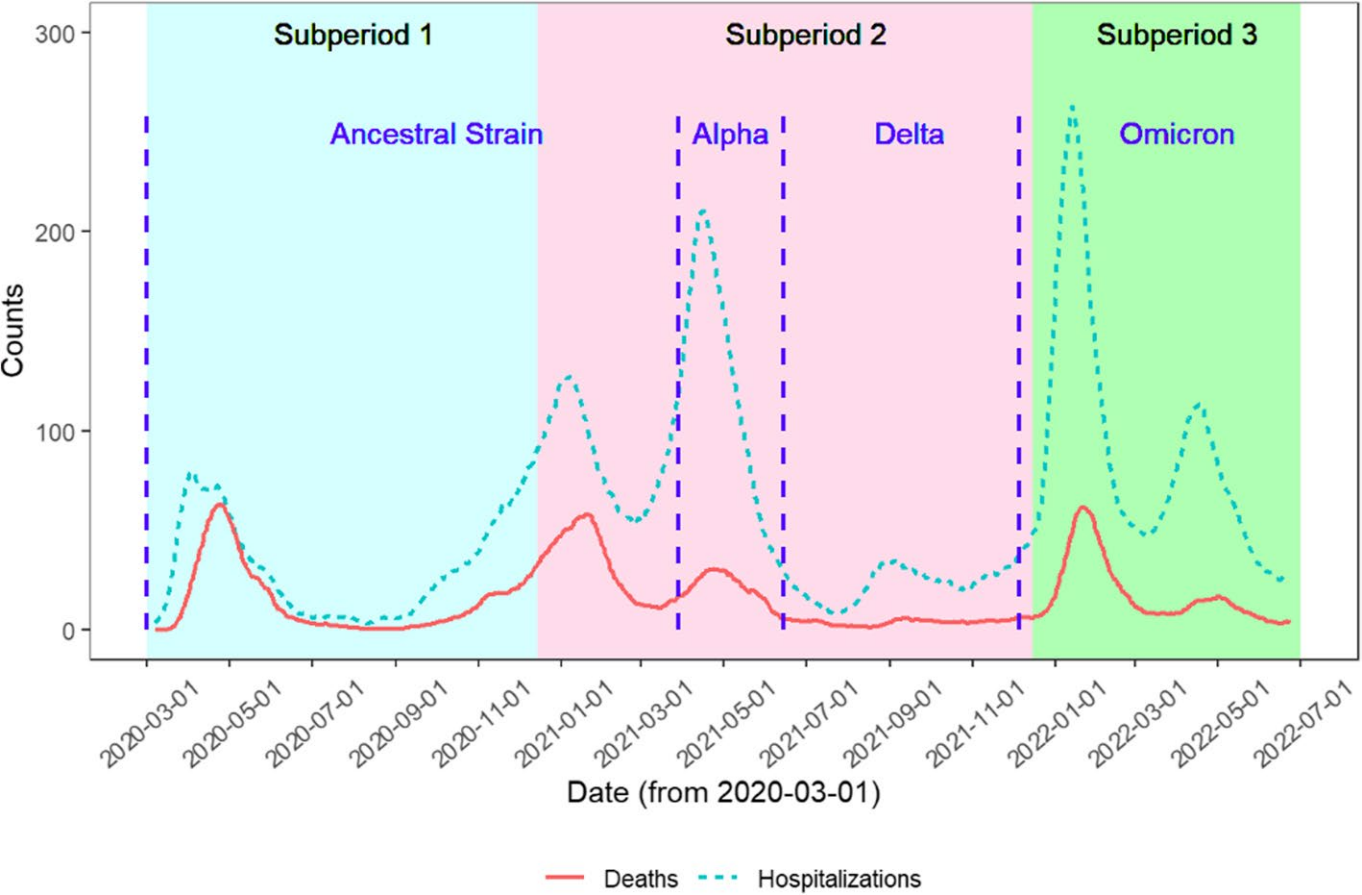
14-day rolling averages of daily deaths and hospitalizations in Ontario, 2020-2022

Figure 2 presents the daily numbers of COVID-19 deaths and hospitalizations in Ontario throughout the study period, with dominant virus variants identified. The shaded regions represent three subperiods and the blue dashed lines indicate the approximate dates when specific variants became dominant (> 50% of cases). We observed the largest daily increase in hospitalizations occurred during the dominance of the Omicron variant, whereas the largest daily deaths were observed during the dominance of the ancestral strain. Additionally, about 80.4% of the daily deaths were zeros over the whole study period. In subperiod 1, the average daily deaths and hospitalizations in Ontario were 14.06 (median: 6.00) and 31.14 (median: 24.00), respectively; in subperiod 2, the average deaths increased to 15.91 (median: 8.00) per day and the average hospitalizations doubled (average: 63.36; median: 40.50). As the Omicron variant spread in Ontario (in subperiod 3), the average daily deaths and hospitalizations further grew to 17.98 (median: 11.50) and 89.13 (median: 64.00).

The rest of the section is organized as follows. In the first three subsections, we present results corresponding to each of the three subperiods, separately. Then, we contrast and summarize those results in the last subsection.

### Results for Subperiod 1 (March 1, 2020 to December 13, 2020)

The final selected model for subperiod 1 is of the form

$$\:\text{l}\text{o}\text{g}\left(\mu_{st}\right)=\beta_0^{\left(1\right)}+b_s^{\left(1\right)}+\beta_1^{\left(1\right)}\text{l}\text{o}\text{g}\left(\text{p}\text{o}\text{p}\text{u}\text{l}\text{a}\text{t}\text{i}\text{o}{\text{n}}_\text{s}\right)+f_1^{\left(1\right)}\left({14\text{d}\text{a}\text{y}.\text{r}\text{o}\text{l}\text{l}\text{i}\text{n}\text{g}.\text{a}\text{v}\text{e}\text{r}\text{a}\text{g}\text{e}.\text{h}\text{o}\text{s}\text{p}\text{i}\text{t}\text{a}\text{l}\text{i}\text{z}\text{a}\text{t}\text{i}\text{o}\text{n}\text{s}}_{st}\right)+f_2^{\left(1\right)}\left(t\right)+f_3^{\left(1\right)}(\text{p}\text{r}\text{o}\text{p}\text{o}\text{r}\text{t}\text{i}\text{o}\text{n}.\text{c}\text{a}\text{s}\text{e}\text{s}.\text{o}\text{v}\text{e}\text{r}60\text{y}\text{r}{\text{s}}_{st}),$$

where $\:s=1,\:\cdots\:,\:34$ and $\:t=1,\:\cdots\:,\:288$. Table 1 shows the estimated linear effects in this selected model. The population size had a positive effect on average daily deaths. When the population size of a health unit doubled in subperiod 1 with other covariates fixed at certain values, the average daily mortality increased by 60.7%.

**Table 1:** GAM Analysis for subperiod 1

Terms	Estimate	Estimated Standard Error	*P*-value
**Linear Effect**			
log(population)	0.684	0.164	< 0.001
**Model**	**Adjusted R-square**	**Deviance explained**	**AIC**
	0.843	81.1%	7277.096

**Fig. 3:**
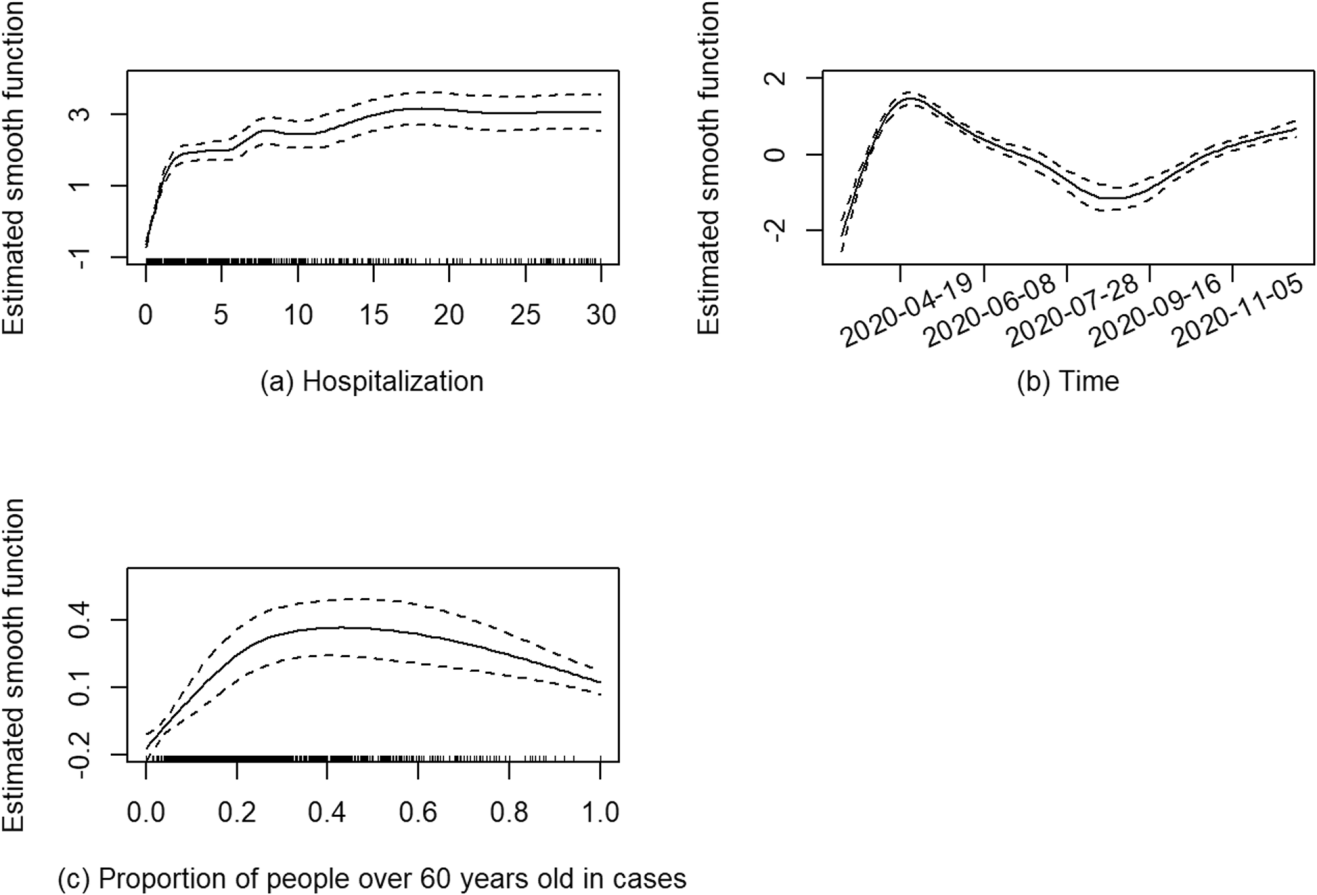
Estimated smooth functions with 95% pointwise confidence intervals (CIs) for subperiod 1

Figure 3 shows the estimated smooth functions with 95% pointwise confidence intervals (CIs); the smooth functions visualize how the logarithm of mean daily COVID-19 mortality changes as one covariate increases with others fixed at certain values. In Fig. 3 (a), the average daily number of COVID-19 deaths initially increased quickly with increasing hospitalizations and levelled off when daily hospitalizations were greater than 15. However, data were sparse at higher numbers of hospitalizations (indicated by the rug along the X-axis of the plot). Figure 3 (b) shows the change in the average daily mortality over time after accounting for the other variables. Specifically, the mean daily deaths rose after March 1, 2020 and peaked around April 24, 2020; thereafter, the count gradually decreased until August 27, 2020, and then began to increase beyond the end of subperiod 1 (December 13, 2020). As illustrated in Fig. 3 (c), the average daily deaths grew quickly as the daily proportion of confirmed cases in subjects over 60 years old, until it reached 0.4, and then levelled off with increasingly sparse data for the proportion greater than 0.6.

**Fig. 4:**
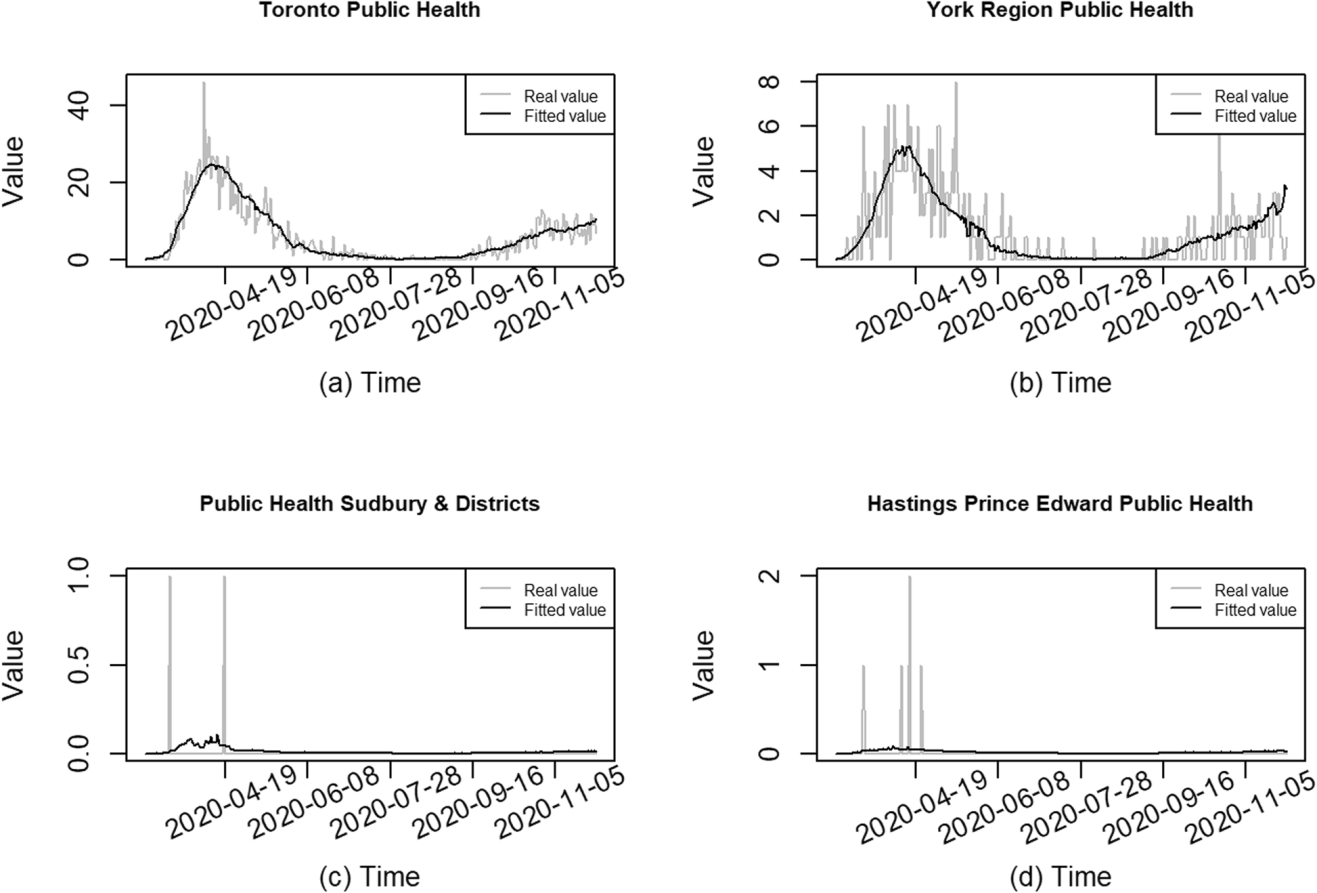
Fitted and observed values of daily deaths for selected health units in subperiod 1

We did not detect obvious violations of model assumptions. See some further model diagnostics in Appendix 2 Fig. S1. We plotted the fitted and observed values of daily deaths in Fig. 4 and found the selected GAM fit the data fairly well for the health units with many daily deaths, such as Toronto and York units, identified in Fig. 4 (a) & (b). In health units with small populations and/or few daily deaths, the model did not fit the data very well, but even so the non-zero death counts (such 1 and 2) occurred at those times near the peak of the fitted values (shown in Fig. 4 (c) & (d) for Sudbury & Districts and Hastings Prince Edward health units). These findings were consistent across all the subperiods.

### Results for Subperiod 2 (December 14, 2020 to December 14, 2021)

The form of the selected model for subperiod 2 is

$$\begin{aligned}\:\text{l}\text{o}\text{g}\left({\mu\:}_{st}\right)={\beta\:}_0^{\left(2\right)}+&b_s^{\left(2\right)}+\beta_1^{\left(2\right)}\text{l}\text{o}\text{g}\left(\text{p}\text{o}\text{p}\text{u}\text{l}\text{a}\text{t}\text{i}\text{o}{\text{n}}_s\right)+{\beta\:}_2^{\left(2\right)}\text{p}\text{r}\text{o}\text{p}\text{o}\text{r}\text{t}\text{i}\text{o}\text{n}.\text{c}\text{a}\text{s}\text{e}\text{s}.\text{o}\text{v}\text{e}\text{r}60\text{y}\text{r}{\text{s}}_{st}\\&+f_1^{\left(2\right)}\left(14\text{d}\text{a}\text{y}.\text{r}\text{o}\text{l}\text{l}\text{i}\text{n}\text{g}.\text{a}\text{v}\text{e}\text{r}\text{a}\text{g}\text{e}.\text{h}\text{o}\text{s}\text{p}\text{i}\text{t}\text{a}\text{l}\text{i}\text{z}\text{a}\text{t}\text{i}\text{o}\text{n}{\text{s}}_{st}\right)+f_2^{\left(2\right)}\left(t\right)\\&+f_3^{\left(2\right)}\left({\text{p}\text{r}\text{o}\text{p}\text{o}\text{r}\text{t}\text{i}\text{o}\text{n}.\text{o}\text{f}.\text{f}\text{u}\text{l}\text{l}\text{y}.\text{v}\text{a}\text{c}\text{c}\text{i}\text{n}\text{a}\text{t}\text{e}\text{d}.\text{o}\text{v}\text{e}\text{r}60yrs}_{st}\right),\end{aligned}$$

where $\:s=1,\:\cdots\:,\:34$ and $\:t=289,\:\cdots\:,\:654$. Table 2 presents the estimated linear effects in subperiod 2. Both the population size and the proportion of reported cases in people over 60 years old had positive effects on the average daily deaths. With other covariates unchanged, the mean daily COVID-19 mortality increased by 11.0% if the population size of a health unit doubled. When other covariates remained the same and the proportion of reported cases in subjects over 60 years old rose by 0.2, the average daily deaths also increased by 11.0%.

**Table 2:** GAM Analysis for subperiod 2

Terms	Estimate	Estimated Standard Error	*P*-value
**Linear Effects**			
log(population)	0.150	0.071	0.034
proportion.cases.over60yrs	0.521	0.153	< 0.001
**Model**	**Adjusted R-square**	**Deviance explained**	**AIC**
	0.793	71.2%	12970.03

**Fig. 5:**
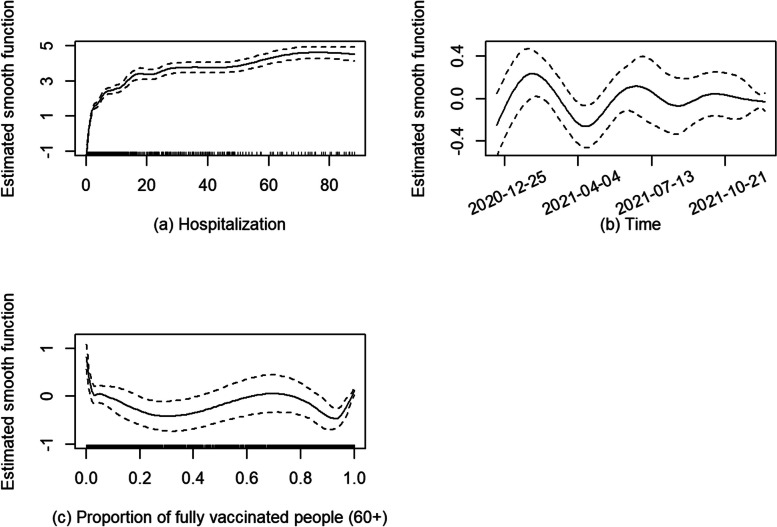
Estimated smooth functions with 95% pointwise confidence intervals (CIs) for subperiod 2

**Fig. 6:**
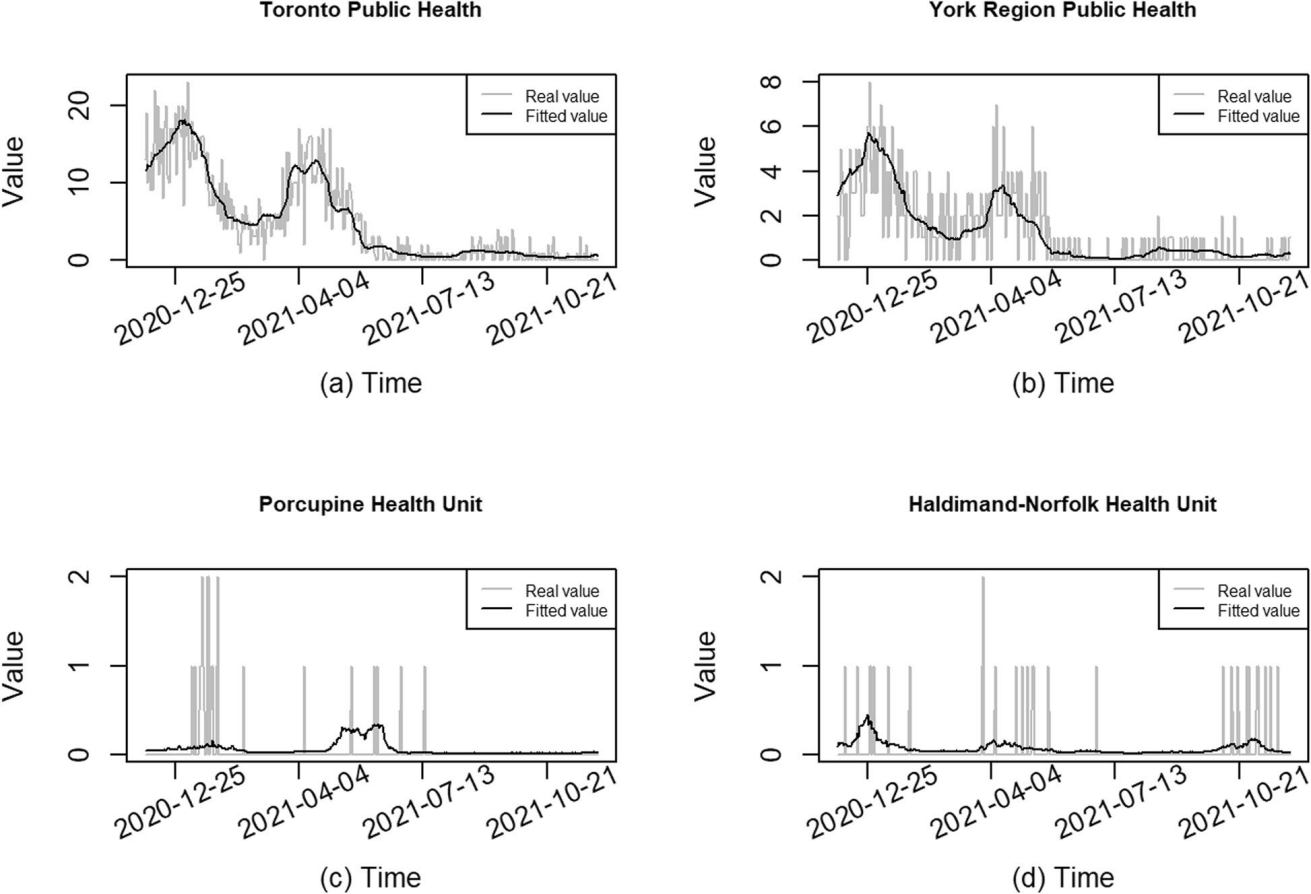
Fitted and observed values of daily deaths for selected health units in subperiod 2

Figure 5 shows the estimated smooth functions in subperiod 2. Figure 5 (a) & (b) can be interpreted similarly to the plots in subperiod 1. However, the X-axis of Fig. 5 (a) acknowledges more daily hospitalizations in subperiod 2 compared with subperiod 1. COVID-19 vaccines became available in Ontario during this subperiod. Figure 5 (c) shows the trend in average daily mortality as the proportion of fully vaccinated people in the 60 + age group increases. Before June 2021, this proportion was below 0.2 in most health units. By July and August 2021, the proportion dramatically increased to more than 0.8. This accounts for the observed narrow confidence intervals in the low and high proportions in Fig. 5 (c). No notable violation of the model assumptions was detected in Appendix 2 Fig. S2. Figure 6 presents the fitted and observed values of daily deaths for selected health units.

### Results for Subperiod 3 (December 15, 2021 to June 30, 2022)

In subperiod 3, the final selected GAM is of the form

$$\begin{aligned} \:\text{l}\text{o}\text{g}\left({\mu\:}_{st}\right)={\beta\:}_0^{\left(3\right)}+b_s^{\left(3\right)}+\beta_1^{\left(3\right)}\text{l}\text{o}\text{g}\left(\text{p}\text{o}\text{p}\text{u}\text{l}\text{a}\text{t}\text{i}\text{o}{\text{n}}_s\right) &+{\beta\:}_2^{\left(3\right)}\text{p}\text{r}\text{o}\text{p}\text{o}\text{r}\text{t}\text{i}\text{o}\text{n}.\text{c}\text{a}\text{s}\text{e}\text{s}.\text{o}\text{v}\text{e}\text{r}60\text{y}\text{r}{\text{s}}_{st}+\beta_3^{\left(3\right)}\text{d}\text{e}\text{p}\text{r}\text{i}\text{v}\text{a}\text{t}\text{i}\text{o}{\text{n}}_s\\&+f_1^{\left(3\right)}\left(14\text{d}\text{a}\text{y}.\text{r}\text{o}\text{l}\text{l}\text{i}\text{n}\text{g}.\text{a}\text{v}\text{e}\text{r}\text{a}\text{g}\text{e}.\text{h}\text{o}\text{s}\text{p}\text{i}\text{t}\text{a}\text{l}\text{i}\text{z}\text{a}\text{t}\text{i}\text{o}\text{n}{\text{s}}_{st}\right)+f_2^{\left(3\right)}\left(t\right)\\&+f_3^{\left(3\right)}\left({\text{p}\text{r}\text{o}\text{p}\text{o}\text{r}\text{t}\text{i}\text{o}\text{n}.\text{o}\text{f}.\text{f}\text{u}\text{l}\text{l}\text{y}.\text{v}\text{a}\text{c}\text{c}\text{i}\text{n}\text{a}\text{t}\text{e}\text{d}.\text{o}\text{v}\text{e}\text{r}60yrs}_{st}\right), \end{aligned}$$

where $\:s=1,\:\cdots\:,\:34$ and $\:t=655,\:\cdots\:,\:852$. Table 3 shows the estimated linear effects in subperiod 3. In addition to the population size and the proportion of confirmed cases in people over 60 years old, deprivation (from the marginalization index) had a positive effect on the average daily mortality. When the deprivation score increased by 0.2, the daily deaths rose by 12.7% on average. Other linear effects can be interpreted as previously. In the dataset, the Halton Region Public Health had the lowest deprivation level at −0.72 (least deprived) and Timiskaming Health Unit had the highest level at 0.55. Statistics Canada [36] described Timiskaming Health Unit as a sparsely populated mix of urban and rural citizens and a moderately high proportion of the population receiving government transfer income. In Timiskaming, 17.3% of total income of private households in 2015 came from government transfers and 21.7% of the population was 65 or older compared to the provincial average of 16.7% [30]. Halton on the other hand had a mainly urban population with a moderate to high population density and a high employment rate [36]. Only 14.9% of the Halton population was aged 65 or older, and 6.7% of total income of private households in 2015 came from government transfers, compared to the provincial average of 11.1% [30]. We will discuss deprivation further in Overall results and Conclusions.

**Table 3:** GAM Analysis for subperiod 3

Terms	Estimate	Estimated Standard Error	*P*-value
**Linear Effects**			
log(population)	0.515	0.068	< 0.001
proportion.cases.over60yrs	0.728	0.207	< 0.001
deprivation	0.596	0.201	0.003
**Model**	**Adjusted R-square**	**Deviance explained**	**AIC**
	0.655	50.4%	9989.852

**Fig. 7:**
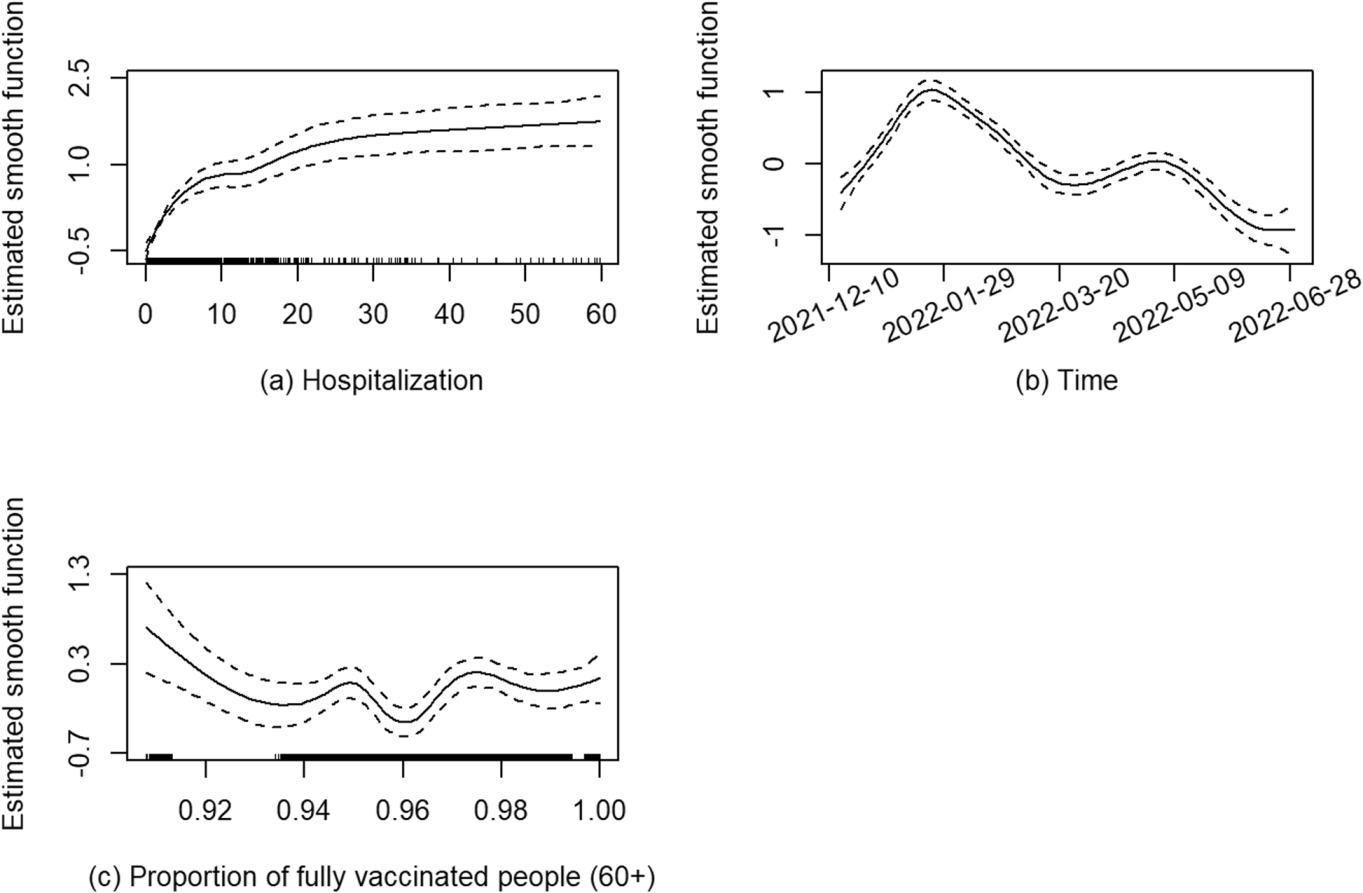
Estimated smooth functions with 95% pointwise confidence intervals (CIs) for subperiod 3

Figure 7 presents the estimated smooth functions in the model of subperiod 3. The smaller magnitude of the Y-axis in Fig. 7 (a) suggests that the daily hospitalizations had a smaller effect on average daily mortality in subperiod 3 compared with those in subperiods 1 & 2. The trend shown in Fig. 7 (b) is consistent with that in Fig. 2. In Fig. 7 (c), the average daily deaths fluctuated as the fully vaccinated proportion in the 60 + age group increased. Since this proportion lies mainly between 0.93 and 0.99 (given most people were already fully vaccinated), we could consider a linear term of the proportion in the model. In the new model, the linear effect of the proportion was not significant. The plots of fitted and observed values of daily deaths and the model diagnoses are shown in Appendix 2 Figs. S3 & S4.

### Overall results

Figures 3(a), 5(a) and 7(a) show the relationship between the logarithm of mean daily deaths and daily new hospitalizations (14-day rolling average) over the three subperiods. In subperiod 1, the mean daily deaths increased by approximately 21 times when the daily hospitalizations increased from 0 to 10 holding other covariates at fixed values. Under the same condition, the mean daily deaths had a 46-fold increase in subperiod 2 and a 4.7-fold increase in subperiod 3. The deprivation had a significantly positive effect on the mean daily deaths only in subperiod 3. There were more public health protections in place during subperiods 1 and 2, which might have reduced the variability in COVID-19 mortality between the advantaged and less advantaged population.

**Fig. 8:**
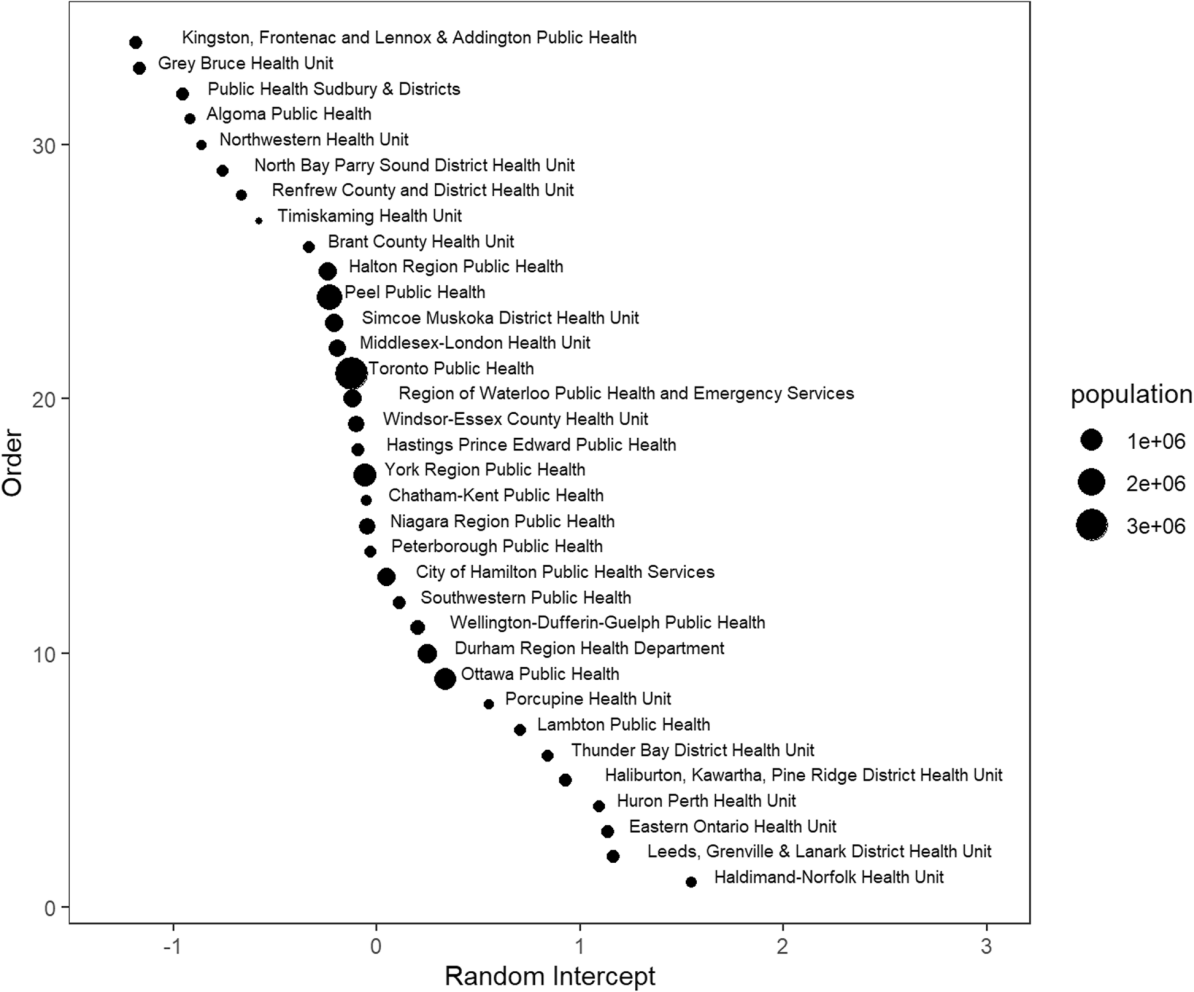
Random intercepts for each public health unit in subperiod 1 (pre-vaccination)

**Fig. 9:**
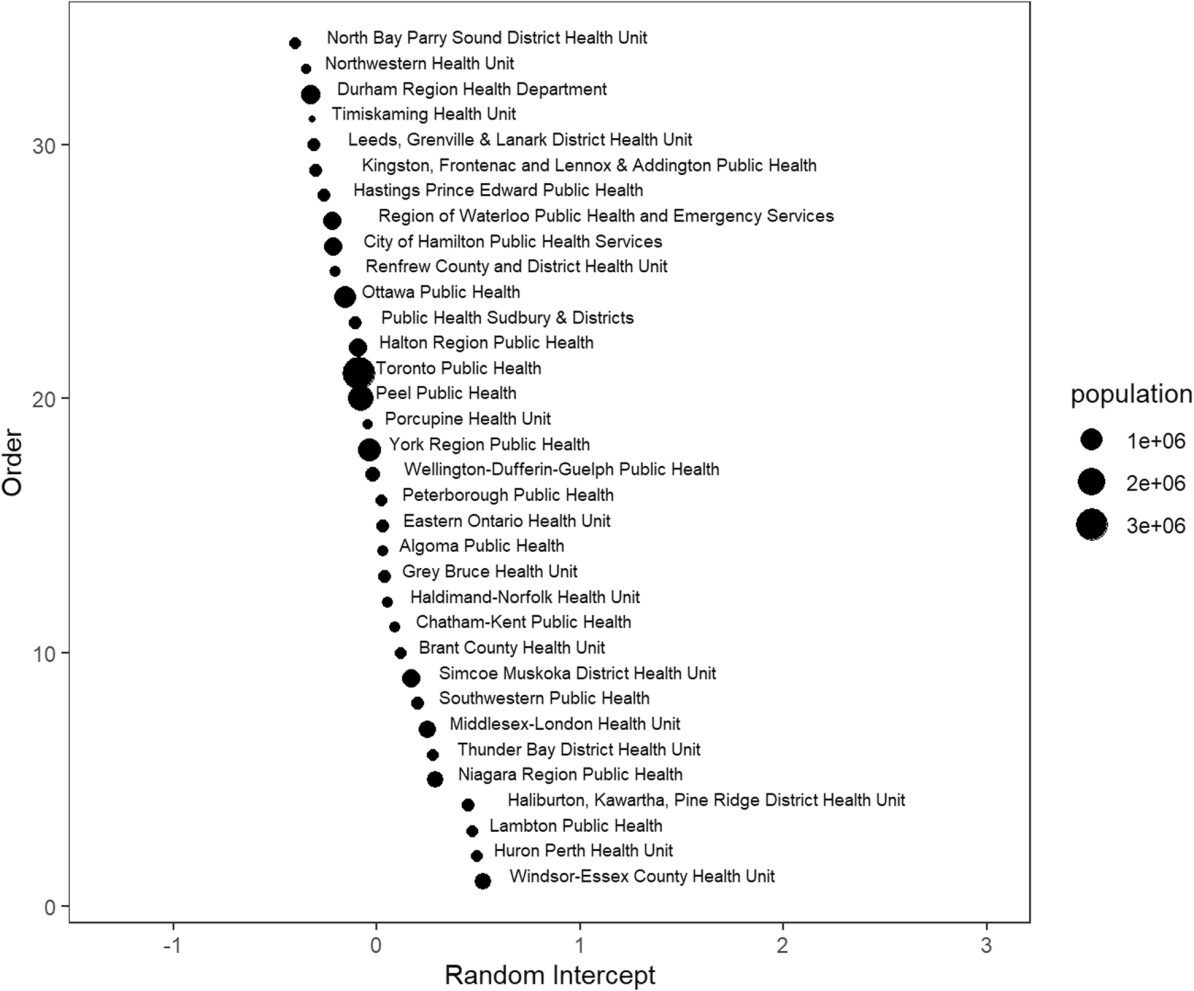
Random intercepts for each public health unit in subperiod 2 (post-vaccination and pre-Omicron)

**Fig. 10:**
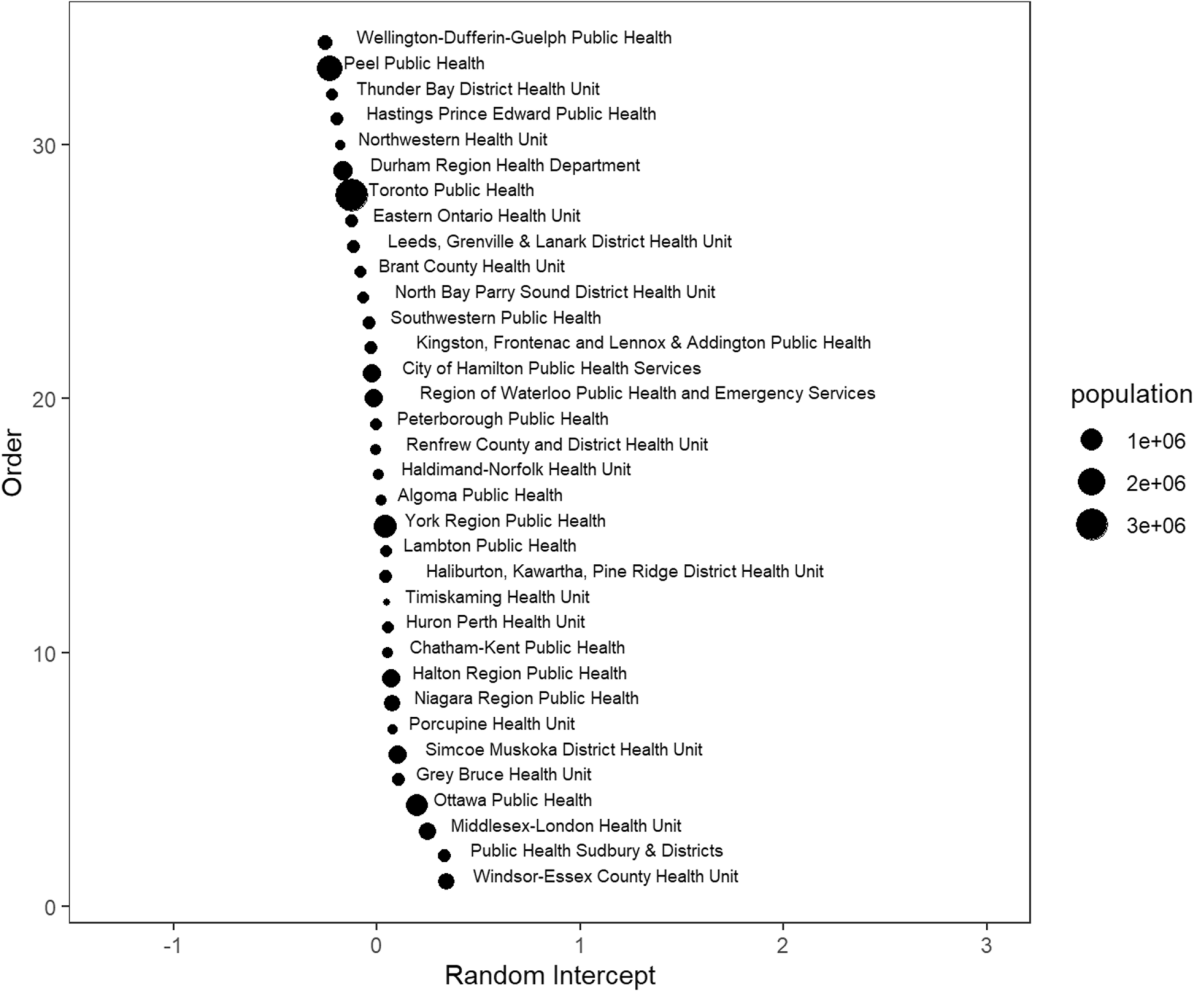
Random intercepts for each public health unit in subperiod 3 (post-Omicron)

Figures 8, 9 and 10 illustrate the random intercepts for health units in each of the three subperiods. The estimated variance of the random intercepts in subperiod 1 is larger than those in subperiods 2 & 3 since the random intercepts in Fig. 8 are more spread out. Possible reasons for the different variances are provided in Discussion. A positive random intercept indicates higher average daily deaths in a specific health unit, given other covariates are fixed. The size of the circle indicates the population size of each health unit. Health units with a large population usually have a random intercept close to zero. We should avoid over-interpreting the rank of the random intercepts, as their order may vary with the model selected for each subperiod.

## Discussion

This study provided regional-level insights into the effects of certain risk factors on COVID-19 related mortality in Ontario, with a focus on the changing dynamics over the three subperiods. The findings reveal heterogeneity in outcomes across regions, driven by various demographic, geographic, socio-economic, and health-related factors. One striking outcome was the continued increase in the average daily deaths and hospitalizations across subperiods, despite the introduction of vaccines. In the early part of subperiod 1, deaths were already high, especially in the large urban centres. Even with the rollout of vaccines in subperiod 2 and the widespread administration of booster does in subperiod 3, we observed that average daily deaths and hospitalizations continued to rise, particularly as the Omicron variant became dominant. This suggests that, while the availability of vaccines reduced severe outcomes, the sheer transmissibility of Omicron contributed to the increasing hospitalizations and deaths especially in the most vulnerable populations such as individuals over 60 years old. The proportion of confirmed cases in people over 60 years old is included as a smooth function only in the selected model of subperiod 1. The linear effect in subperiod 3 is larger than that in subperiod 2, likely due to widespread transmission after lifting of most public health interventions. The effect of population size on mean daily deaths was significant, particularly in subperiod 1, where health units with larger populations experienced higher mortality rates. This aligns with global patterns, where urban centres with larger, denser populations faced higher transmission rates and consequently worse outcomes. The degree of socio-economic marginalization likely exacerbated the burden in the larger cities, highlighting that underlying health inequities made some populations more susceptible to severe outcomes.

Examining specific health units, the highest mortality rates were observed in Toronto and in health units with the busiest border crossings to the United States. Prior to vaccine availability, there was a greater variance in the health units, as shown in Fig. 8, suggesting that some characteristics of the administrative health regions might influence outcomes. This may warrant further investigation. The random intercepts in Figs. 8, 9 and 10 show a changing pattern across the subperiods, with the most variation in subperiod 1 and the least in subperiod 3. This reduced variation in subperiod 3 may be due to consistency of policies across health units, the lifting of health protections, and the rising level of population immunity from both vaccination and prior infections. Some points that stand out from Figs. 8, 9 and 10 include that Haldimand Norfolk (HDN) had the largest random intercept in subperiod 1, remaining the largest throughout the study period. Four health units - HDN, Huron Perth (HPH), Lambton (LAM), and Haliburton Kawartha Pine Ridge (HKPR) - maintained consistently positive random intercepts across all three subperiods. Many factors may contribute to a higher random intercept for deaths; however, we note that all four units have a notably larger proportion of population aged 65+, exceeding the provincial average of 16.7%, with HKPR having the largest at 26.4%, and all units exceeding 20%. In contrast, eight health units consistently ranked with negative random intercepts, including Peel (PEL), Hastings Prince Edward (HPE), Northwestern (NWR), Toronto (TOR), North Bay Parry Sound (NBPS), Kingston Frontenac Lennox and Addington (KFLA), Waterloo (WAT), and Renfrew (REN). Of these, four units (PEL, NWR, TOR, and WAT) have a lower proportion of population over aged 65 compared to the provincial average. The remaining health units (HPE, NBPS, KFLA, and REN) are sparsely populated, mainly rural (with some rural-urban mix) regions, with populations aged 65 + ranging from 20 to 23% [30]. The availability of vaccination could help reduce differences in COVID-19 related mortality between health units. However, despite vaccination, when public health protections were removed, the least advantaged might suffer higher COVID-19 mortality. Interventions such as paid sick days and cleaner indoor air should be made available to counter lifting of health protections.

### Limitations

In this study, the data are presented at the level of public health unit, which can vary dramatically in population density, geographic size, and urban vs. rural composition. There is also much variation within each health unit. The data may not be granular enough for meaningful conclusions at the unit-level. The GAMs performed well in regions with higher daily death counts, such as Toronto and York, but they struggled in smaller health units with low daily death counts (≤ 2). This likely reflects the challenges involved in modeling sparse and zero heavy data. Future research could explore alternative approaches to better capture this variability in regions with low mortality rates and small population size.

We applied a GAM to quantify the association of deaths with hospitalizations across the 34 public health units in Ontario in each subperiod, while taking other covariates into account. The daily count of COVID-19 deaths in each health unit was overdispersed and had a large proportion of zeros. We tested both a negative binomial family and a zero-inflated Poisson family and found that the model in a negative binomial family had a smaller AIC value and a larger adjusted R-square and/or deviance explained. We assumed that observations were independent in time and geographic locations, however the number of deaths was potentially temporally and spatially correlated. A future investigation taking the spatio-temporal correlation into account would work better with individual-level COVID-19 data.

## Conclusions

We have shown that a GAM is a valuable method for examining risk factors associated with COVID-19 mortality. We found that it was important to stratify time over the study period since there were different public health policies in place, with variability in implementation, as well as different variants of SARS-CoV-2 circulating. We focused on the confirmed cases and the vaccination rates in the 60 + age group, where most deaths occurred. Extending our analysis to all age groups did not result in a significant improvement in the model fit. However, this finding may change with a longer follow up period, as vaccination coverage expanded across all age groups.

The overall pattern of daily deaths in Ontario closely follows daily hospitalizations throughout all three subperiods (Fig. 2). The increase in *overall* average daily deaths and hospitalizations in Ontario over the last two subperiods is likely due to a combination of the lifting of many public health protections along with the increased transmissibility of the Omicron variant [37]. Additionally, a low death rate among infected individuals and a high infection rate with the advent of Omicron can lead to an increase in the number of COVID-19 deaths. The overall effect of hospitalizations on deaths becomes smaller in subperiod 3, as indicated by the smaller magnitude of the Y-axis in the estimated smooth function for subperiod 3 (Fig. 7(a)) compared to subperiods 1 and 2.

The deprivation measure from the marginalization index is a measure of poverty – the higher deprivation score, the worse economic situation. This measure has a significantly positive effect on mortality only in subperiod 3, which suggests that the least advantaged are most affected by COVID-19-related mortality when the fewest protections are in place. The lack of significance in subperiods 1 and 2 could be related to broadly applied public health interventions on the least deprived population during these two subperiods. These broad public health measures helped protect disadvantaged groups or groups affected by higher levels of deprivation, which might have limited their access to vaccines. Keep in mind that deprivation levels vary *within* each health unit; the deprivation scores in our dataset provide an overall picture of each health unit in Ontario. This finding does highlight the importance of targeted interventions in more deprived areas during a public health crisis. The significant effect of deprivation on mortality in subperiod 3 suggests that as broad protections were reduced, socioeconomic disparities became more pronounced. Long-term solutions require addressing underlying social determinants of health to ensure equitable outcomes across all communities.

Differences in COVID-19 mortality between subperiods may be associated with several factors not captured in our models, such as stringency of public health interventions, travel and border restrictions, and features of viral variants. In subperiod 1, public health interventions were stronger and consistent across all health units. In subperiod 2, public health interventions were allowed to vary by health unit for the first half of the subperiod. In subperiod 3, public health protections initially in place were consistent across health units. However, health protections were lifted by the end of February 2022, with mask mandates ending in March 2022; during the bulk of subperiod 3, there were few public health protections in place besides wearing masks by personal choice and in health care settings. Our findings emphasize the need for targeted interventions in densely populated and socio-economically marginalized areas. These regions may benefit from ongoing surveillance and resource allocation in terms of public health campaigns to better protect vulnerable populations. Additionally, the overall increase in deaths and hospitalizations during the Omicron period suggests that vaccines alone may not be sufficient to fully mitigate the impact of highly transmissible variants. Continued booster campaigns and public health protections such as masking and cleaner indoor air may still be necessary. Ongoing vigilance and adaptive public health strategies will be essential to address the upcoming challenges posed by new variants and continuing disparities in health outcomes.

## Supplementary Information

Supplementary Material 1.

## Data Availability

The dataset analyzed during the current study is no longer publicly available due to updates on Public Health Ontario repository website but are available from the corresponding author on reasonable request. We used publicly available census data from Statistics Canada, which has an open licence available here: https://www.statcan.gc.ca/en/reference/licence. This license agreement allows the use of Statistics Canada information without restrictions on sharing and redistribution, for commercial and non-commercial purposes.

## Abbreviations

AIC: Akaike information criterion
CI: Confidence interval
COVID-19: Coronavirus disease
GAM: Generalized additive model
GLM: Generalized linear model
HDN: Haldimand Norfolk
HKPR: Haliburton Kawartha Pine Ridge
HPE: Hastings Prince Edward
HPH: Huron Perth
KFLA: Kingston Frontenac Lennox and Addington
LAM: Lambton
LASSO: Least absolute shrinkage and selection operator
NBPS: North Bay Parry Sound
NIA: Niagara Regional Area
NWR: Northwestern
PEL: Peel
PHO: Public Health Ontario
REN: Renfrew
SARS-CoV-2: Severe acute respiratory syndrome coronavirus 2
SE: Standard error
TOR: Toronto
WAT: Waterloo
WEC: Windsor-Essex County

## References

[1] Bialek S, Boundy E, Bowen V, Chow N, Cohn A, Dowling N, et al. Severe outcomes among patients with Coronavirus Disease 2019 (COVID-19) — United States, February 12–March 16, 2020. MMWR Morbidity Mortal Wkly Rep. 2020;69(12):343–6. 10.15585/mmwr.mm6912e2 PMC7725513 32214079

[2] World Health Organization. Estimating mortality from COVID-19. World Health Organization; 2020. https://www.who.int/news-room/commentaries/detail/estimating-mortality-from-covid-19. Accessed 30 Nov 2024.

[3] Acosta AM, Garg S, Pham H, Whitaker M, Anglin O, O’Halloran A, et al. Racial and ethnic disparities in rates of COVID-19–associated hospitalization, intensive care unit admission, and in-hospital death in the United States from March 2020 to February 2021. JAMA Netw Open. 2021;4(10):e2130479. 34673962 10.1001/jamanetworkopen.2021.30479 PMC8531997

[4] Borgonovi F, Andrieu E, Subramanian SV. The evolution of the association between community level social capital and COVID-19 deaths and hospitalizations in the United States. Soc Sci Med. 2021;278:113948. 33930677 10.1016/j.socscimed.2021.113948 PMC8055504

[5] Cade BE, Dashti HS, Hassan SM, Redline S, Karlson EW. Sleep apnea and COVID-19 mortality and hospitalization. Am J Respir Crit Care Med. 2020;202(10):1462–4. 32946275 10.1164/rccm.202006-2252LE PMC7667903

[6] Kim L, Garg S, O’Halloran A, Whitaker M, Pham H, Anderson EJ, et al. Risk factors for intensive care unit admission and in-hospital mortality among hospitalized adults identified through the U.S. coronavirus disease 2019 (COVID-19)-associated hospitalization surveillance network (COVID-NET). Clin Infect Dis. 2020. https://pubmed.ncbi.nlm.nih.gov/32674114/. Accessed 30 Nov 2024. 10.1093/cid/ciaa1012 PMC7454425 32674114

[7] Price-Haywood EG, Burton J, Fort D, Seoane L. Hospitalization and mortality among black patients and white patients with covid-19. N Engl J Med. 2020;382(26). https://www.nejm.org/doi/10.1056/NEJMsa2011686. Accessed 30 Nov 2024. 10.1056/NEJMsa2011686 PMC7269015 32459916

[8] Chaudhry R, Dranitsaris G, Mubashir T, Bartoszko J, Riazi S. A country level analysis measuring the impact of government actions, country preparedness and socioeconomic factors on COVID-19 mortality and related health outcomes. EClinicalMedicine. 2020;0(0). https://www.thelancet.com/journals/eclinm/article/PIIS2589-5370(20)30208-X/fulltext. Accessed 30 Nov 2024. 10.1016/j.eclinm.2020.100464 PMC7372278 32838237

[9] Pan A, Liu L, Wang C, Guo H, Hao X, Wang Q, et al. Association of public health interventions with the epidemiology of the COVID-19 outbreak in Wuhan, China. JAMA. 2020;323:19. 32275295 10.1001/jama.2020.6130 PMC7149375

[10] Talic S, Shah S, Wild H, Gasevic D, Maharaj A, Ademi Z, et al. Effectiveness of public health measures in reducing the incidence of covid-19, SARS-CoV-2 transmission, and covid-19 mortality: systematic review and meta-analysis. BMJ. 2021;375:8315. 10.1136/bmj-2021-068302 PMC9423125 34789505

[11] Dam D, Merali S, Chen M, Coulby C, Ho B, Bang F, et al. COVID-19 outcome trends by vaccination status in Canada, December 2020–January 2022. Can Commun Dis Report. 2024;50(1/2):40–8. 10.14745/ccdr.v50i12a05 PMC11037879 38655240

[12] Tenforde MW, Self WH, Adams K, Gaglani M, Ginde AA, McNeal T, et al. Association between mRNA vaccination and COVID-19 hospitalization and disease severity. JAMA. 2021;326(20):2043–54. 34734975 10.1001/jama.2021.19499 PMC8569602

[13] Little C, Alsen M, Barlow J, Naymagon L, Tremblay D, Genden E, et al. The impact of socioeconomic status on the clinical outcomes of COVID-19; a retrospective cohort study. J Community Health. 2021;46:794–802. 33387149 10.1007/s10900-020-00944-3 PMC7775835

[14] Esper FP, Adhikari TM, Tu ZJ, Cheng YW, El-Haddad K, Farkas DH, et al. Alpha to Omicron: disease severity and clinical outcomes of major SARS-CoV-2 variants. J Infect Dis. 2023;227(3):344–52. 36214810 10.1093/infdis/jiac411 PMC9619650

[15] Mendiola-Pastrana IR, López-Ortiz E, Río de la Loza-Zamora JG, González J, Gómez-García A, López-Ortiz G. SARS-CoV-2 variants and clinical outcomes: a systematic review. Life. 2022;12(2):170. 35207458 10.3390/life12020170 PMC8879159

[16] Profiles of public health systems in Canada: Ontario. https://ccnpps-ncchpp.ca/docs/2021-Profiles-of-Public-Health-Systems-in-Canada-Ontario.pdf. Accessed 30 Nov 2024.

[17] Allan M, Lièvre M, Laurenson-Schaefer H, de Barros S, Jinnai Y, Andrews S, et al. The World Health Organization COVID-19 surveillance database. Int J Equity Health. 2022;21:21(S3). 36419127 10.1186/s12939-022-01767-5 PMC9685131

[18] Blauer B, Brownstein J, Gardner L, Moritz Ug Kraemer, Zoila Leiva Rioja, Mathieu E, et al. Innovative platforms for data aggregation, linkage and analysis in the context of pandemic and epidemic intelligence. 2023;28(24). https://www.ncbi.nlm.nih.gov/pmc/articles/PMC10318939/. Accessed 30 Nov 2024. 10.2807/1560-7917.ES.2023.28.24.2200860 PMC10318939 37318761

[19] Khan D, Park M, Burkholder J, Dumbuya S, Ritchey MD, Yoon P, et al. Tracking COVID-19 in the United States with surveillance of aggregate cases and deaths. Public Health Reports. 2023;138(3):428–37. 36960828 10.1177/00333549231163531 PMC10040484

[20] Couture A, Iuliano D, Chang H, Patel N, Gilmer M, Steele M, et al. Estimating COVID-19 hospitalizations in the United States with surveillance data using a bayesian hierarchical model: a modeling study (preprint). JMIR Public Health Surveill. 2022;8(6):e34296. 35452402 10.2196/34296 PMC9169704

[21] Fulcher IR, Boley EJ, Gopaluni A, Varney PF, Barnhart DA, Kulikowski N, et al. Syndromic surveillance using monthly aggregate health systems information data: methods with application to COVID-19 in Liberia. Int J Epidemiol. 2021;50(4):1091–102. 34058004 10.1093/ije/dyab094 PMC8195038

[22] Lima EEC de, Gayawan E, Baptista EA, Queiroz BL. Spatial pattern of COVID-19 deaths and infections in small areas of Brazil. PLoS One. 2021;16(2):e0246808. 33571268 10.1371/journal.pone.0246808 PMC7877657

[23] Karmakar M, Lantz PM, Tipirneni R. Association of social and demographic factors with COVID-19 incidence and death rates in the US. JAMA Netw Open. 2021;4(1):e2036462. 33512520 10.1001/jamanetworkopen.2020.36462 PMC7846939

[24] Hastie T, Tibshirani R. Generalized additive models: some applications. J Am Stat Assoc. 1987;82(398):371–86.

[25] Ramsay T, Burnett R, Krewski D. Exploring bias in a generalized additive model for spatial air pollution data. Environ Health Perspect. 2003;111(10):1283–8. 12896847 10.1289/ehp.6047 PMC1241607

[26] Ravindra K, Rattan P, Mor S, Aggarwal AN. Generalized additive models: building evidence of air pollution, climate change and human health. Environ Int. 2019;132:104987. 31398655 10.1016/j.envint.2019.104987

[27] Feng C, Kephart G, Wagner BD. Predicting COVID-19 mortality risk in Toronto, Canada: a comparison of tree-based and regression-based machine learning methods. BMC Med Res Methodol. 2021;21(1):1–14. 34837951 10.1186/s12874-021-01441-4 PMC8627169

[28] Feng C. Spatial-temporal generalized additive model for modeling COVID-19 mortality risk in Toronto, Canada. Spat Stat. 2022;49:100526. 34249608 10.1016/j.spasta.2021.100526 PMC8257405

[29] Izadi F. Generalized additive models to capture the death rates in Canada COVID-19. In: Springer eBooks. 2021. p. 153–71. 10.1007/978-3-030-85053-1_7.

[30] Statistics Canada. Census profile, 2016 census. 2021. https://www12.statcan.gc.ca/census-recensement/2016/dp-pd/prof/details/page.cfm?Lang=E&Geo1=CD&Code1=3554&Geo2=PR&Code2=35&SearchText=Timiskaming&SearchType=Begins&SearchPR=01&B1=All&TABID=1&type=0. Accessed 30 Nov 2024.

[31] Public Health Ontario. Ontario COVID-19 data tool. Public Health Ontario. 2021 [cited 2023 Aug 1].

[32] Ontario Marginalization Index (ON-Marg). Public Health Ontario. https://www.publichealthontario.ca/en/data-and-analysis/health-equity/ontario-marginalization-index. Accessed 6 Jul 2023.

[33] Tøge AG, Bell R. Material deprivation and health: a longitudinal study. BMC Public Health. 2016;16(1):747–747. 27501962 10.1186/s12889-016-3327-z PMC4977874

[34] Public Health Ontario. Easy maps tool. https://www.publichealthontario.ca/en/Data-and-Analysis/Commonly-Used-Products/Maps/Easy-Map/Easy-Map-Full. Accessed 6 Jul 2023.

[35] 511 Ontario. Traveller information. 2022. http://www.mto.gov.on.ca/english/ontario-511/ontario-border-crossings.shtml. Accessed 6 Jul 2023.

[36] Statistics Canada. Health Region Peer Groups – working paper, 2018. 2018. https://www150.statcan.gc.ca/n1/pub/82-622-x/82-622-x2018001-eng.htm#fn. Accessed 6 Jul 2023.

[37] Public Health Ontario. Recommendations: fourth COVID-19 vaccine dose for long-term care home residents and older adults in other congregate settings. 2021. https://www.publichealthontario.ca/-/media/documents/ncov/vaccines/2022/01/covid-19-oiac-4th-dose-recommendations-older-adults-ltc.pdf?sc_lang=en. Accessed 6 Jul 2023.

