# Supplementary material for: The effects of deprivation, age, and regional differences in COVID-19 mortality from 2020 to 2022: a retrospective analysis of public provincial data
Source: BMC Public Health. 2025 Jan 14;25:148. doi: 10.1186/s12889-024-21031-5 (PMC11730143; doi:10.1186/s12889-024-21031-5)
Supplement: Supplementary file 1 — Supplementary Material 1. [file 12889_2024_21031_MOESM1_ESM.docx]

**Appendix 1: Implementation of the GAMs** **in R**

The function *bam* in the *mgcv* R package was used to fit the GAMs. The *bam* function took less computing time and occupied less memory than the conventional function *gam* for handling such a large dataset. The double penalty approach was applied by setting *bs=”cr”* (i.e., the penalized cubic regression splines) for smooth functions and *select=T* in the *bam*. The function *gam.check* validated whether the selected number of basis functions was large enough for a specific smooth function. The sum-to-zero constraint was applied as the identifiability constraints of the smooth functions; that is, the sum of a smooth function over all observed values of the covariate was equal to zero. We included the logarithm of the population size as a covariate rather than an offset, which allowed the estimated coefficient to be different from 1, giving a more flexible GAM and an opportunity for a differential handling of how population size affected the outcome over the three subperiods. We used the restricted maximum likelihood (REML; *method= "REML"* in the *bam*) to estimate the smoothing parameters because REML is more robust to under-smooth (overfitting) and obtains less minima than generalized cross-validation approaches (GCV). All the analyses were conducted in R (Version 4.0.2).

**Appendix 2: Additional Plots**

Fig. S1 shows the diagnostic plots generated by the R function *gam.check()*, which are used to access model fit and check model assumptions for subperiod 1. In the upper-left plot, most points lie roughly along a straight line, indicating no major violation against our distribution assumption. In the residuals vs. linear predictor plot, the deviance residuals are not randomly scattered as the linear predictor increases, but this is acceptable given that the response variable is counts. The histogram of the deviance residuals shows a left tail, consistent with the positive departures seen in the upper-left plot. However, considering the discrete nature of the response, we do not view this as a severe violation of the model assumptions. Lastly, the plot of response vs. fitted values presents a positive linear relationship, with no notable issue observed. Fig.s S2 and S4 can be interpreted similarly.


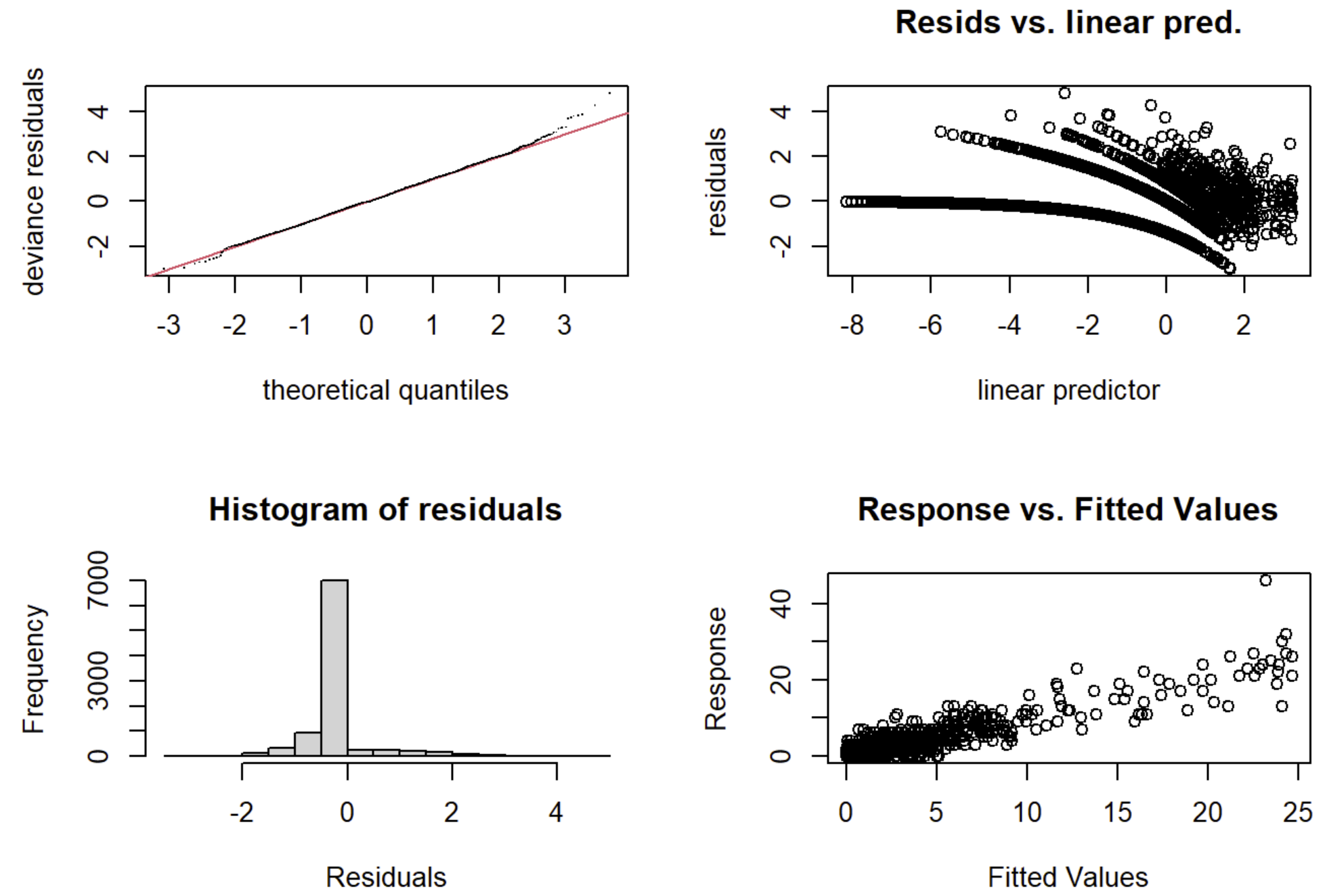


**Fig. S1** Model diagnostics for the GAM in subperiod 1


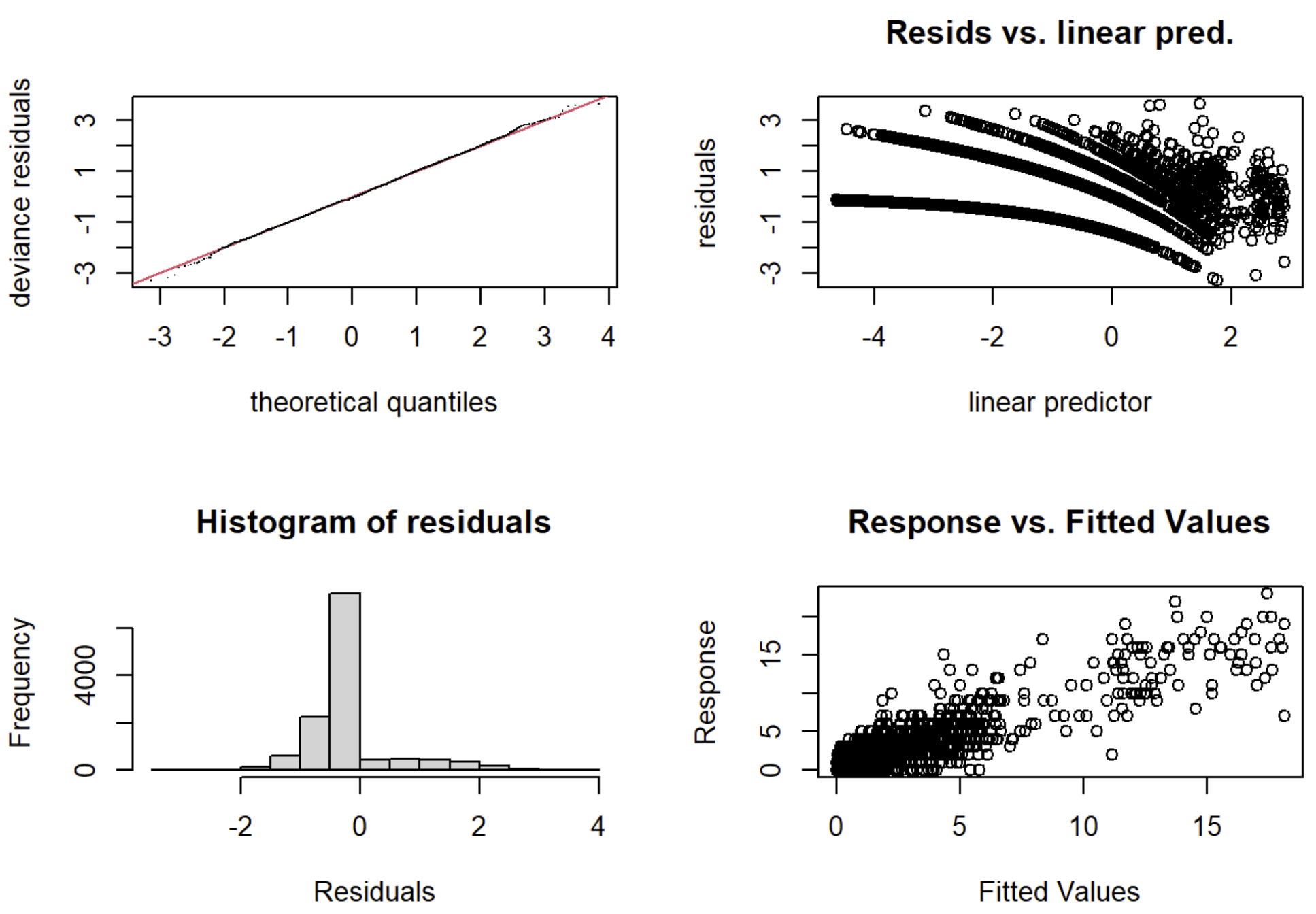


**Fig. S2** Model diagnostics for the GAM in subperiod 2

**
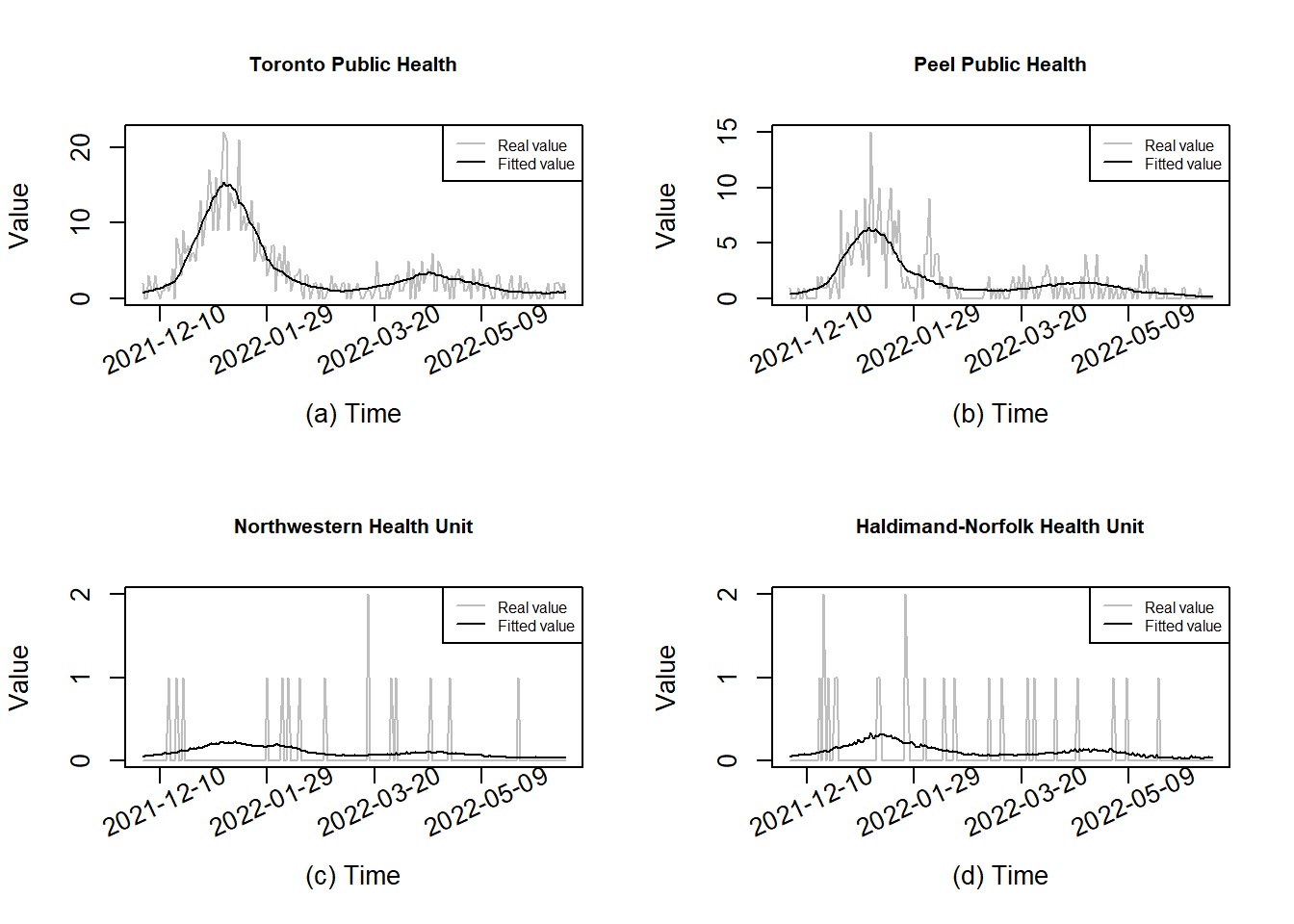
**

**Fig. S3** Fitted and observed values of daily deaths for selected health units in subperiod 3


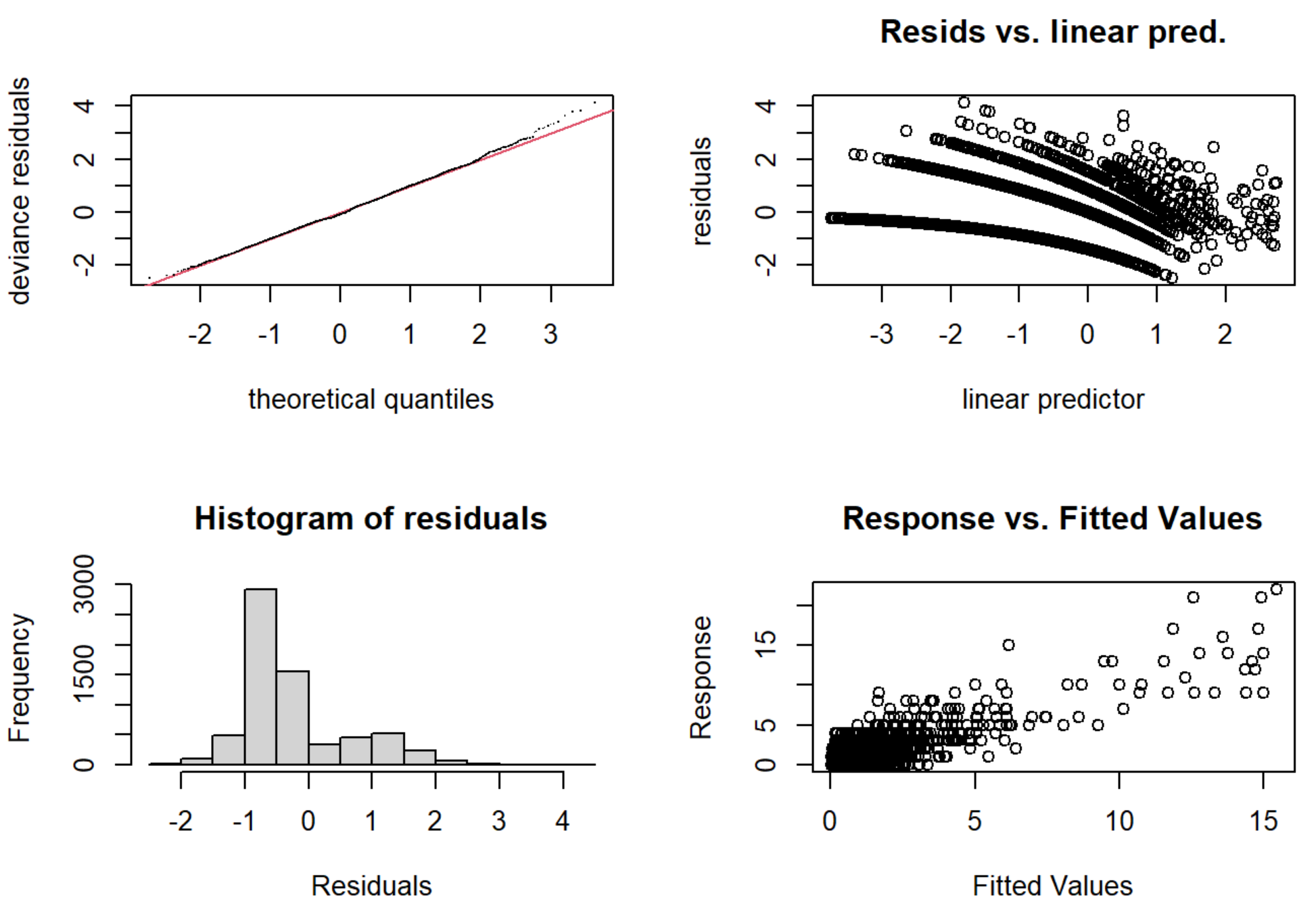


**Fig. S4** Model diagnostics for the GAM in subperiod 3
